# Deformed nail from stress related to childbearing

**DOI:** 10.1002/jgf2.283

**Published:** 2019-10-07

**Authors:** Ryuichi Ohta

**Affiliations:** ^1^ Community Care Unnan City Hospital Unnan Japan

**Keywords:** Beau's line, malformed, nail diseases, nails, pregnancy

## Abstract

Deformed nail from stress related to childbearing with a 36‐year‐old woman (a late phase of Beau's line).

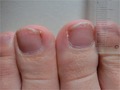

A 36‐year‐old postdelivery woman presented at a community hospital with a chief complaint of nail deformities in bilateral feet, with a 9‐month‐old boy. There were no other symptoms. Physical examination was normal, except for nail deformities, known as Beau's line, in bilateral feet (Figure 1). This deformity involves transverse depressions in the nail that result from any episodic disease that is severe enough to disrupt the normal nail growth, such as diseases due to chemotherapeutic agents and abrupt onset of systemic diseases^1^. Based on her clinical course, the cause of Beau's line was suggested to be the stress of childbearing, which is a rare case. As the transverse depressions on the nails were not typical of Beau's line, this was thought to correspond to the late phase of the condition. The length of the normal part of her nails (growth rate of foot nails: approximately 0.05 mm/day) indicated that her childbearing approximately 160 days prior might have been the cause of the deformity^2^. She recalled the toughness of her childbearing caused by stress, which resulted in sleep interruption and appetite loss when her child was 0‐4 month old. Her clinical course was excellent, and all her nails were clear at the 3‐month follow‐up, with no treatments.

**Figure 1:**
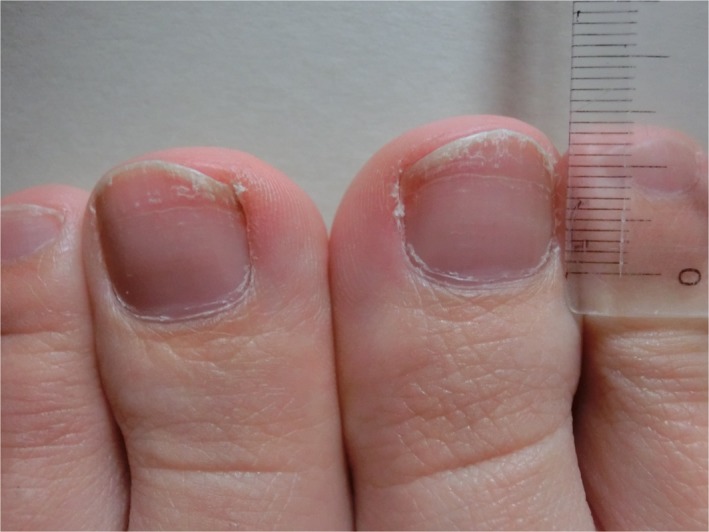
Beau's line in bilateral feet

## CONFLICT OF INTEREST

None declared.

## PATIENT CONSENT

Patient consent was obtained before publishing this case report.

## References

[1] Fawcett RS , Linford S , Stulberg DL . Nail abnormalities: clues to systemic disease. Am Fam Physician. 2004;69(6):1417–24. 15053406

[2] Yaemsiri S , Hou N , Slining MM , He K . Growth rate of human fingernails and toenails in healthy American young adults. J Eur Acad Dermatol Venereol. 2010;24(4):420–3. 19744178 10.1111/j.1468-3083.2009.03426.x

